# A qualitative study of GP, nurse and practice manager views on using targeted case-finding to identify patients with COPD in primary care

**DOI:** 10.1038/s41533-017-0049-3

**Published:** 2017-08-29

**Authors:** Rachael H. Summers, Taniya Sharmeen, Kate Lippiett, Kate Gillett, Carla Astles, Linh Vu, Mark Stafford-Watson, Anne Bruton, Mike Thomas, Tom Wilkinson

**Affiliations:** 1 Faculty of Health Sciences, University of Southampton, Southampton, SO17 1BJ UK; 2 School of Pharmacy, University Reading, Reading, RG6 6UA UK; 3 NIHR CLAHRC Wessex, Faculty of Health Sciences, University of Southampton, Southampton, SO17 1BJ UK; 4 University Hospital Southampton NHS Trust, Southampton, SO16 6YD UK; 5 Psychology, University of Southampton, Southampton, SO17 1BJ UK; 6 NIHR Southampton Biomedical Research Centre, University Hospital Southampton NHS Trust, Southampton, SO16 6YD UK; 7 Primary Care and Population Sciences, University of Southampton, Southampton, SO16 5ST UK; 8 Clinical and Experimental Sciences, University of Southampton, Southampton, SO16 6YD UK

## Abstract

‘Finding the missing millions’ with chronic obstructive pulmonary disease became part of the Department of Health strategy for England in 2010. Targeted case-finding within primary care is one potential pro-active strategy, but currently little is known about the views of healthcare professionals on this approach. In this study, 36 healthcare professionals (12 GPs, 14 nurses, and 10 practice managers) from 34 UK practices participated in semi-structured telephone interviews about targeted case-finding. Interviews followed an interview guide, were audio-recorded, transcribed verbatim, coded and analysed using ‘Framework Approach’. Most of those interviewed practiced opportunistic case-finding. The main perceived barriers to wider case-finding programmes were the resource implications associated with running such programmes and identifying more chronic obstructive pulmonary disease patients. Financial incentives, support from specialist clinicians, and comprehensive guidance were viewed as facilitators. While targeted case-finding is conceptually accepted by primary care staff, scepticism surrounding (1) the value of identifying those with mild disease and (2) the availability of effective targeted case-finding methods, may lead some to favour an opportunistic approach. Key concerns were a lack of unequivocal evidence for the relative benefits vs. disadvantages of diagnosing patients earlier, and resource constraints in an already over-burdened system. Barriers to practical implementation of case-finding studies may be addressed with financial, human and educational resources, such as additional staff to undertake searches and perform spirometry tests, and practical and educational support from specialist teams.

## Chronic obstructive pulmonary disease: support needed to identify those undiagnosed

Additional staff and resources would facilitate targeted searches for patients showing symptoms of early-stage chronic lung disease. Chronic obstructive pulmonary disease (COPD) costs the UK economy billions of pounds each year, yet disparate symptoms mean patients aren’t always diagnosed in the early, treatable stages of the disease. Recent guidelines suggest introducing ‘targeted case-finding’, where symptomatic patients with known risk factors are identified and approached for testing by doctors. Rachael Summers and colleagues at the University of Southampton analyzed the opinions of healthcare professionals on implementing targeted case-finding in primary care. While most of the 36 professionals interviewed agreed that diagnosing COPD earlier had clear benefits, concerns were raised regarding negative patient responses and increased stress for patients, alongside the added strain on already stretched resources. Employing independent staff and enhancing resources may facilitate such a program.

## Introduction

Chronic obstructive pulmonary disease (COPD) costs the United Kingdom (UK) economy £2.7 billion per year in lost working days and costs the NHS £800 million per year.^1, 2^ The greatest cost is associated with hospital admissions for those with severe/very severe COPD.^1^ Early diagnosis of COPD has the potential to reduce these costs, because although COPD cannot be cured, it is treatable once a diagnosis has been made.^3^ Early diagnosis is, therefore, a key tenet of the UK’s national strategy for managing COPD.^4^

In the UK, it is estimated that 2 million people with COPD remain undiagnosed,^5^ with a diagnosis often not being made until disease is relatively advanced.^6^ There are several reasons for this delay in diagnosis. As the disease usually has an insidious onset, patients may initially attribute symptoms to those expected of normal ageing, or from smoking, and so avoid seeking medical advice.^7^ A general lack of public awareness about COPD may also contribute to late presentation.^8^ Evidence also suggests that opportunities for diagnosis in primary care may be missed, with many patients presenting with COPD-related symptoms (such as cough and breathlessness) being misdiagnosed as simple respiratory tract infections, rather than acute exacerbations of COPD. Jones et al.^6^ retrospective analysis of 38,859 patient cases revealed that in the 5 years immediately before diagnosis, there were missed diagnostic opportunities in 85% of patients.

Two related approaches for diagnosing COPD available are population-based screening (testing all at high risk including asymptomatic patients), and targeted case-finding (testing those with risk factors, symptoms or health resource utilisation patterns suggestive of COPD). Screening asymptomatic at-risk populations is not currently supported by the evidence,^9, 10^ but identifying COPD in symptomatic patients is advocated^3, 10^ and supported by research.^11^ Case-finding for COPD may be either targeted (where symptomatic patients with known risk factors are systematically identified) or opportunistic (where symptomatic patients with known risk factors are identified when they attend for routine healthcare consultations).^12^ Haroon et al.^13^ identified a number of approaches for COPD case finding in primary care. Approaches included targeted and opportunistic case-finding. Screening questionnaires, handheld metres to identify airflow obstruction and direct invitations for diagnostic spirometry were used either in as combined or as standalone approaches. However relatively little is known about the views on case-finding of those working in primary care, who have to administer the case-finding programme and deal with the consequences of additional newly diagnosed patients. Historically, clinicians have been reported to hold nihilistic attitudes towards the diagnosis of COPD,^14^ so understanding their views and identifying facilitators and barriers to implementation is likely to be important to conducting successful case-finding initiatives. Case-finding initiatives have implications for the entire primary healthcare team, including non-clinical staff, and understanding their concerns and motivations is important.

While there have been clinician commentaries on the value of case-finding^15, 16^ and the general importance of COPD in primary care has been explored,^17^ only Haroon et al.^18^ qualitative study has specifically explored primary care staff views on case-finding. While this study makes an important contribution, finding interest in case-finding despite concerns over financing and capacity, it was restricted to practices in the West Midlands, involved only three practice managers, and all practices were already participating in a trial of case-finding. This may have affected the views expressed, as those included probably already had an interest in case-finding. Further research into general practice staff’s views involving a sample independent of a case-finding trial is needed to increase our understanding of how case-finding is viewed in the wider primary care staff population. This study aimed to explore the views of GPs, nurses and practice managers on targeted case-finding for COPD within general practices in the South of England.

## Results

A total of 42 people from 37 practices responded to the initial contact (15 GPs, 17 Nurses, and 10 Practice Managers). Of those initially responding, 36 staff (12 GPs, 14 Nurses, and 10 Practice Managers) (Table 1) from across 34 GP practices (Table 2) were interviewed. Of those not interviewed: (i) four did not respond to follow-up contact, (ii) one withdrew before the interview for personal reasons, and (iii) one nurse was not interviewed as s/he was not demographically dissimilar to other participants in the nurse sample, and data saturation was judged to have been reached. Data were organised into two themes:

**Table 1:** Participant characteristics

Characteristics	General Practitioner	Nurse	Practice manager
Staff interviewed (n = 36)	12	14	10
Age range in years (Mean)	32–60(45)	38–59(49)	34–54(42)
Sex			
Female	5	14	9
Male	7	0	1
Length of practice experience			
<5 years	2	1	5
6–10 years	4	6	1
>10 years	6	7	4
COPD lead	5	9	N/A
Nurse role			
Practice Nurse	N/A	13	N/A
Nurse Practitioner		1	
Partnership in the practice	11	N/A	N/A

**Table 2:** Practice characteristics

Characteristic	Number
Total number of practices	34
Practice list sizes	
Range	3670–37,000
<5000	3
5000–10,000	11
>10,000	20
Practice type	
NHS	1
Independent	33
Practice location	
Urban	18
Suburban	11
Rural	5

*Positions on diagnosing COPD*: contains views on COPD diagnosis in terms of its importance both generally and at a practice level, and perceived advantages and drawbacks.

*Views on approaches to COPD case-finding*: summarises data relating to views regarding the value and desirability of different case-finding strategies, the relative pros and cons of targeted case-finding, and interviewees’ self-reported willingness to participate in externally run targeted case-finding initiatives.

### Positions on diagnosing COPD

All the participants considered diagnosing COPD to be important. Advantages ascribed to diagnosing COPD included: giving patients the knowledge, and possibly motivation, to make positive lifestyle changes; ensuring medical management was optimal, thereby enhancing patient quality of life; and saving NHS resources through reduced admissions for exacerbations and other problems associated with COPD.

I think that’s an important thing to do and obviously by stopping the decline in lung function, we can keep patients better, more well for longer, and also reduce the impact the disease has not only on themselves but on the NHS in terms of exacerbations, prescriptions involving expensive medication. [GP 10102, Male]

However, clinician views differed regarding the ‘best’ time to diagnose COPD (while practice managers did not discuss this). A majority of GPs and nurses (23/26) argued that diagnosing COPD as early as possible was key, including those with mild COPD.

if you found them around the 80 per cent or just below the 80 per cent when they had much milder damage and they stopped smoking, they exercised, they’d got their flu and pneumonia jabs, looked after themselves, it would make such a lot of difference in the long run [Practice Nurse 19215, Female]

Three GP interviewees were unconvinced as to the value of diagnosing mild COPD and/or those who were asymptomatic. While these GPs considered diagnosing people with mild COPD to be beneficial if it resulted in smoking cessation, no other benefits to diagnosing COPD in the mild stage were perceived. Furthermore, they viewed the consequence of having to see those with mild/asymptomatic COPD for annual review as placing unnecessary strain on already stretched services. A practice manager, who described proactively diagnosing cases (of any condition) earlier as “a luxury”, epitomised this view.

Whether it’s cancer or COPD or dementia or whatever […] it’s lovely to be able to pick up a case earlier, but you’ve got to have the resource up-front to be able to invest in picking that up, and the NHS is so stretched at the moment, that you can’t afford the luxury of it. Because that’s what it is, really: it’s a luxury. [Practice Manager 26307, Female]

if people come to you and you give them a diagnosis of mild COPD, I think you’ve got to question what exactly we’ve got to offer them. Like I say, is it a case of diagnosing them, they still smoke and they want to change and take a step to stop smoking, that’s very good […] but given that we’re only going to start them on symptomatic management, if they’re not feeling symptomatic I’m not sure what benefit it is in just telling them they’ve got mild COPD. [GP 33113, Male]

Potential drawbacks to diagnosing COPD were noted across all interviews. It was recognised that, for patients, receiving a COPD diagnosis could entail stress, worry and stigmatisation. People also commented on the personal financial ramifications of diagnosing someone with COPD, such as increased insurance costs, adverse effects on an individual’s occupation, and unnecessary medicalisation. The financial implications for both running case-finding initiatives and managing greater numbers of people with COPD were of particular concern.

Time. […] I’m also looking after all the asthmatics. I’m also expected to do travel vaccinations, B12s, sorting immunisation, you name it. It’s a time implication isn’t it? So if I end up with [100 more] patients with COPD, [who] I’ve got to see a minimum of 30 min a year, usually more than that, let’s be honest. So the more patients you have, the more people you’ve got to see on a regular basis [Practice Nurse 07207, Female]

All interviewees described the challenge of trying to balance providing best care and meeting clinical need with limited resources. COPD was contextualised within a larger picture, as one condition amid many others requiring GP practice attention. There were two reported priorities at practice level: (1) fulfilling local clinical need and (2) meeting UK clinical targets such as the ‘National Quality and Outcomes Framework (QOF)’ (an incentive scheme in England offering financial rewards to GP practices meeting specified clinical targets) and the local targets set by clinical commissioning groups (CCGs).

**Interviewer:** So what influences this prioritisation?

**Practice Manager 11301:** There are two parts to this, really. One of them is clinical need, based on the extent of the problem, and the other one, although it sounds quite bad to say it, is financial. […] for a practice to earn its money, we have to achieve a certain number of points which are related to the quality and outcomes framework QOF enhanced service. So we try to balance it out across all of the areas so that everything is dealt fairly, but sometimes if you have a low prevalence in one particular area and it’s higher in the other, it is more—I don’t like using this word, but it’s more profitable to concentrate on the areas where you have the higher prevalence […] But as I said, that has to be balanced with clinical need and the need of the patient.

The mix of nationally (i.e., QOF) and locally (i.e., CCG) defined targets may explain variance between interviewee accounts as to how COPD was prioritised at practice level.

I think it’s whatever comes through the door. So if somebody comes through the door with problems with breathing, it’s a priority to discover what is causing that, rather than actually prioritising COPD in itself. [Practice Nurse 14210, Female]

Well, the chronic disease, the QOF has been a big drive, so they prioritise the clinic highly with the COPD, the diabetes or the other QOF clinics, as it were, cardiovascular disease. [Practice Nurse 21211, Female]

### Views on approaches to COPD case-finding

Interviewees were largely supportive of case-finding as a concept. Most clinicians and practice managers reported opportunistic case-finding to be their practice’s usual approach, although a small number used both opportunistic and targeted strategies. Many were interested in the idea of using electronic searches to undertake targeted case-finding, viewing this as a potentially useful approach; some even considered targeted case-finding to be the ‘gold standard’, with opportunistic strategies being less adequate.

you know, I think that—not just for COPD, but I think for anything, that’s [targeted case-finding] fantastic, and that’s obviously the gold standard. [Practice manager 6307, Female]

That’s how I would like to work, where you’re targeting the people, the actual patients that may well have COPD but haven’t yet presented for anything to do with COPD. I think it would be so much better to be able to do that rather than the opportunistic. [Practice Nurse 29213, Female]

However, some concerns surrounding the practicalities of targeted case-finding were highlighted (Table 3). It was noted that, as clinician recording of patient problems/management in electronic records could be idiosyncratic, some questioned the reliability of electronic searches (See 3.1, Table 3). Targeted case-finding was associated with increased resource demand compared to opportunistic case-finding (See 3.2, Table 3). Whilst some reported interest in engaging in targeted case-finding irrespective of how it was resourced, many reported being under considerable pressure to meet existing practice demands. A key consideration was how targeted case-finding would impact on staff time.

I think as long as it was resourced […] I think many people would be very interested but it would need to be resourced, either with a research nurse or equivalent funds to do that. I think trying to recycle it out of current workload would be very challenging [GP 32112, Male]

**Table 3:** Concerns surrounding targeted case-finding

Explanation	Example quotation
3.1 Staff use different terms/approaches to electronic documentation (‘read coding’) within and between practices—so reliability of electronic searches may be poor	*Ideally if you were to do case finding you’d do it with a computer system where everybody is using the same read codes and using them regularly. Unfortunately that doesn’t exist in or out of hospital or community care […] read coding is not a very exact science, everybody that does it does things very differently in their own practice so it’s very hard to consistently pick up the right people. We found actually that we were probably just as successful by doing opportunistic screenings than by actually going through the notes and trawling for read codes and people who are presenting with certain things. [GP10102, Male]*
3.2 Short term resource implications (staffing, time and space)	*The cost [of targeted case-finding]. […] Someone to search the people, someone to call the people, the nurse then to see them, the healthcare support worker to do the spirometry, etcetera, etcetera, and as I said, it’s difficult. We’re finding it very difficult in our practice at the minute anyway for appointments’ (Nurse Practitioner29216, Female)*
3.3 Long term resource implications (maintaining income)	*“I suppose one of the issues […] is the QOF scenario, where if you suddenly code all these people as having COPD, QOF would like them to have an annual review. They’d like them to have the regular spirometry, […] particularly in a small practice like myself, […] even if the initial work had been done in the case-findings, the diagnoses and the education and things, it might be difficult. […] and that’s [QOF generate] the sort of money that pays for the nurses, the annual health checks and things.”[GP33113, Male]*
3.4 Patient candour and uptake of case-finding invitations	*“trying to also persuade people to come in for screening can be difficult. […] I get this list and I ring these patients up to try and persuade them to come in, and they make excuses. There are some that are very happy to come in, but there are some that are quite resistant, and I think they worry that you’re going to tell them off because they’re smokers” [Practice Nurse06206, Female]*

Such accounts emphasised the need for targeted case-finding to be adequately resourced. Providing externally funded personnel to perform the searches or undertake the spirometry was described as one means of potentially facilitating practice engagement. The perceived benefits associated with this related not only to protecting the time of practice staff, but also as a potential opportunity for educational development. Financially incentivising case-finding searches or rewarding practices for identifying new cases of COPD was another suggestion. Other practical considerations such as room space and equipment were also advanced as issues for consideration.

Provided the time and resource, […] and if you can wrap it up with some education then it’s a win for the practice in terms of education. […] make sure that the practice sees that both them and their patients are getting something positive out of it and it isn’t going to be too time consuming. [GP 23109, Male]

Another issue related to the potential longer-term financial consequences of identifying more people with COPD (See 3.3, Table 3). These concerns paralleled those raised in theme 1, including: (1) increases to the practice’s prescription expenditure, (2) increased expenditure for secondary care referral, and (3) decreases to practice income if the practice failed to fulfil QOF criteria for COPD. All of these were described as potential barriers to engagement with targeted case-finding.

A key finding was that not all interviewees thought targeted case-finding was worthwhile, with some considering opportunistic case-finding to be the best approach. Two GPs and one practice manager expressed this view, though their explanations differed slightly. One GP considered the issues with electronic record-keeping (previously described) to render electronic searching too unreliable. The other GP argued that opportunistic case-finding was more likely to yield a population amenable to behaviour change, in contrast to targeted case-finding, which he considered more likely to identify those who were not ready for advice or intervention.

I think that this opportunistic case-finding is definitely the best approach to adopt. I don’t think that extending that into patients who don’t perceive themselves to have a problem is anywhere near as productive. [GP 08101, Male].

This issue was expressed in other interviews to a lesser degree, however, those interviewees remained open to targeted case-finding, provided searches targeted those who were symptomatic or with a number of high risk factors.

Interviewees raised several patient-related factors (See 3.4, Table 3) that they considered would affect the success of targeted case-finding, such as low patient turnout rate for screening appointments, or patients providing incorrect information on life-style choices (like smoking behaviour and diet). However, whilst such issues were mentioned by several interviewees, only one felt that these issues would actually deter their practices from being involved in a case-finding study; this was related to the practice having experienced difficulty when attempting targeted case-finding previously.

## Discussion

### Main findings

There was general support for case-finding amongst this group of nurses, GPs and practice managers. Most were very interested in the idea of targeted case-finding, but this interest was tempered by concerns that targeted case-finding could have limited effectiveness due to problems with electronic record coding, and that the additional resources required to implement targeted case-finding would have both short-term and long-term financial implications. Within these accounts, there was also concern that identifying many more people with COPD than there was capacity to review would lead to a failure to meet financial (QOF) targets; this led some to view targeted case-finding with caution and some misgivings. Targeted case-finding was not viewed favourably by all interviewees; a small number considered opportunistic case-finding to be preferable to targeted case-finding, as the latter was perceived as more likely to identify asymptomatic people, or those with mild COPD (for whom the benefit of early identification was seen as questionable). Lack of patient engagement in screening programmes was also put forward as a barrier to implementation, with some interviewees having reservations over participating in future targeted case-finding after having experienced low uptake in previous case-finding initiatives.

### Interpretation of findings in relation to previously published work

Our findings echo some of those presented by Haroon et al.,^18^ reinforcing their likely salience to Primary Care. Like Haroon et al.,^18^ we found that clinicians generally supported case-finding and that opportunistic case-finding was the most common description of usual practice. However, while many of our interviewees felt early diagnosis was desirable, others questioned the benefit of diagnosing mild COPD. Arguments against diagnosing mild COPD were that: (1) this group (who were considered unlikely to be experiencing noticeable deficits) would be less amenable to changing behaviour, such as smoking; (2) intervention for mild COPD would not make a substantial difference; (3) the cost associated with the increased number of review appointments needed, and additional interventions or referrals, would outweigh the benefits of early diagnosis. Whilst such views were not reported in Haroon et al.^18^ study, Walters et al.^14^ previously identified ‘nihilistic’ attitudes to diagnosing COPD amongst GPs in primary care. Yet, unlike Walters et al.^14^ research, clinicians in this sample were not against diagnosing COPD in general, only in mild or asymptomatic cases where the impact of the condition was not thought considerable enough to motivate behaviour change or have a significant impact on outcomes. The debate around whether early diagnosis is beneficial is not new. In 1999, White^16^ published a commentary arguing against Thomas and Levy^15^ published work, which had recommended increasing early diagnosis of COPD. Today, there is discourse supporting early diagnosis,^4, 19, 20^ but our study reflects the ongoing potential for uncertainty amongst clinicians about the value of early diagnosis of COPD. Clinicians who remain unconvinced will require evidence of benefit for early diagnosis, underscoring the need for further research assessing the impact of diagnosing mild COPD, and the impact of early intervention.

There was a frequently expressed need for additional resources and support to enable practices to engage in targeted case-finding initiatives and to cater for those patients identified as a result, as reported by Haroon et al.^18^ This is perhaps unsurprising given the considerable increase in demand for primary care appointments^21^ and the general staffing and resource challenges currently facing GP practices.^22, 23^ Like Haroon et al.^18^ participants, our interviewees were concerned that their already over-burdened practices would not have capacity to meet the needs of a patient population expanding due to an increase in diagnosed COPD; with capacity to perform yearly reviews being of particular concern.

Another finding was concern about the potential for incurring financial losses as a result of failing to meet QOF targets, which could act as a deterrent to GP practice engagement. Conversely, some interviewees welcomed identifying more people with COPD within their practices, believing this to be beneficial for both improving patient care and generating QOF related revenue for the practice. To our knowledge, the importance of QOF, whilst evident in this study, has not been explicitly identified in earlier work into case-finding for COPD,^18^ though previous literature has raised the issue of QOF shaping GP practice behaviours in other areas.^24–26^

### Strengths and limitations of this study

This study was implemented following a careful, methodologically robust design. Multiple coding and peer review from our multidisciplinary research team was used to critically analyse the data collected. Whilst we were unable to obtain a purposive sample, the sample contained GP practice staff from practices of varying demographics (list sizes, urban vs. rural) from regions in the south of the UK (Wiltshire, Dorset, Hampshire) and was not associated with an ongoing case-finding study. Our participants, who were diverse in relation to age, sex, years of primary care experience and special interest in COPD, provided insights into case-finding for COPD; for this reason, we do not believe that recruitment limitations unduly affected our findings.

### Implications for future research, policy and practice

Further research investigating the cost-benefit of identifying and managing mild/asymptomatic COPD is needed. Strategies to support practices to meet their population demands and any additional resource or fiscal implications of identifying more people with COPD are needed. As such, research to identify clinically and cost effective case-finding approaches is needed. This will need to include long term follow-up to fully understand the impact of case-finding in relation to disease progression, clinical outcomes and socioeconomic cost. This study has highlighted professionals’ concerns over patient engagement in screening programmes; further research is therefore needed to understand patient uptake of case-finding appointments to identify (1) to what extent patients in primary care engage in case-finding and (2) what factors facilitate or inhibit such engagement.

## Conclusions

While primary care HCPs in this study generally supported case-finding and the need to diagnose COPD, views were mixed regarding the value of targeted case-finding and identifying those with mild/asymptomatic COPD. Key concerns were lack of evidence for the effectiveness or benefit of targeted case-finding vs. perceived potential disadvantages to patients, and concerns over financial implications and need for additional resources. The successful implementation of targeted case-finding for COPD in primary care faces a number of barriers. Our findings suggest that one way to enhance practice engagement with targeted case-finding initiatives or studies would be to provide additional administrators and clinicians to support targeted searches, and to perform the spirometry tests needed to diagnose COPD.

## Methods

A qualitative semi-structured interview design was used to capture participant views. The study was designed and implemented using a subtle realist, pragmatic approach. By taking a subtle realist position, we tried to capture a truthful account of the research topic, while recognising that only a best approximation of truth may ever be obtained.^27^ A pragmatic approach meant we selected data collection and analysis strategies appropriate to the research aim, without reference to a particular theoretical lens.^28^

Ethical approval for this study was granted by the University of Southampton Research Ethics Committee (Ethics no. 12647) and methods were performed in accordance with relevant regulations/guidelines. GPs, practice managers, and practice nurses involved in the management of COPD (or in the triage of patients with acute exacerbations of COPD), working in a primary care practice at the time of the study, were eligible to participate.

We aimed to obtain a diverse sample including staff from urban, suburban and rural practices with a range of patient list sizes, with varying years’ experience in primary care and/or managing COPD. We sought GPs who were respiratory leads and those who were not, salaried GPs and GP partners. Interviews were planned to continue until data saturation had occurred, where subsequent interviews ceased to provide new information,^28^ or up to 45 participants (*n* = 15 from each group) had been recruited.

As a diverse sample was sought, the study was advertised as widely as possible. Study recruitment packs (study invitation, information sheet and brief demographics form) were sent via email to the practice manager of 99 GP practices in Wessex; practice managers were asked to disseminate the study invitation amongst all GP and nursing staff. The study was also advertised at a North Hampshire Primary Care Meeting (*n* = 10 practices in attendance), via social media and the Respiratory Primary Care Education Database. Those interested in participating returned their demographics form by email or hard copy and were contacted by the research team to discuss the study in more detail and to establish eligibility. Snowballing was used to supplement recruitment where it would add diversity to the demographic profile of the sample, or the perspectives already captured within. Written consent was obtained from all participants prior to a telephone interview. A £50 incentive was offered to participants to cover time and inconvenience.

### Data collection

Participants took part in a telephone interview, conducted by TS, a Research Fellow with experience of utilising qualitative in methods. Prior to study commencement, participants were previously unknown to TS and no information about the researcher was provided to participants. Interviews were audio-recorded digitally, and followed a staff specific interview guide (Table 4) which was informed by relevant literature,^14, 18^ developed by RS, KL, KG, CA, MSW, AB, MT and TW, and refined following peer review by two independent Research Fellows with expertise in qualitative methods. Interviews ranged from 24 to 79 min (median 41:22 min). Audio-recordings were transcribed verbatim by a professional transcriber and checked by TS for accuracy. Initial interview transcripts from each staff group were circulated among RS, KL, KG, CA, and TW, who provided peer review and advice, prior to further interviews.

**Table 4:** Interview guide questions

The following outlines the main interview questions. Please note, for brevity, only the chief interview question is included below, prompts have been removed.
Staff specific interview guide questions
*Questions for nurses*
• What do you think the role of the nurse is in the management of COPD?
• Have you suggested to a GP that a diagnosis of COPD might be appropriate for a particular patient or assisted in making a diagnosis of COPD? If yes, what lead you to believe they had COPD?
*Questions for GPs*
• What do you think the role of the GP is in the management of COPD?
• What would make you suspect a patient has COPD?
*Questions for Practice Managers*
• What do you think the role of the practice manager is in primary care?
Questions for all staff groups
• In relation to diagnosing COPD, how well do you think the UK is doing?
• Some figures suggest 2 million more people in the UK may have COPD than are currently diagnosed. How important is it to identify people with COPD?
• What do you think about the idea of case-finding as a way of identifying people with COPD?
• If we were to run case-finding in GP practices in the future, how interested do you think practices would be to participate?

### Data analysis

Data were managed using NVivo 10 software and analysed using Framework Approach,^29^ which consisted of five analytic steps:

1. Familiarisation: the first five GP, nurse and practice manager transcripts collected were repeatedly read to identify recurrent concepts and ideas.
2. Preliminary thematic framework development: recurrent concepts identified during the familiarisation process are grouped into initial themes.
3. Indexing: the preliminary thematic framework was then applied consistently to all the transcripts. As indexing continued, the preliminary framework was refined to ensure the framework adequately captured views expressed within the interviews.
4. Charting: each segment of raw data indexed within the framework was summarised.
5. Mapping: That the range of opinions across the data, and the similarities and differences within the data, were described.

TS acted as primary analyst, analysing all data. Other authors (RS, KL, KG, CA, LV, MSW, AB, MT, TW) provided multiple coding and peer review at points throughout the analysis to enhance rigour, with RS, a Senior Research Fellow with doctoral and post-doctoral experience of qualitative methods, supervising and coordinating analysis. Team meetings to discuss the data analysis were held regularly; where coder/peer reviewer interpretation of the data differed from the primary analyst, differences were discussed and resolved by team consensus. Participant checking was not used.

### Data availability

All data and internal working documents generated and used during this study are held at the University of Southampton and are available from the corresponding author upon reasonable individual access to document request according to University of Southampton policy and NHS Research Ethics Committee approval.
